# Evaluating a Middle-School Digital Citizenship Curriculum (Screenshots): Quasi-Experimental Study

**DOI:** 10.2196/26197

**Published:** 2021-09-15

**Authors:** David S Bickham, Summer Moukalled, Heather K Inyart, Rona Zlokower

**Affiliations:** 1 Digital Wellness Lab Division of Adolescent and Young Adult Medicine Boston Children's Hospital Boston, MA United States; 2 Media Power Youth Manchester, NH United States

**Keywords:** digital citizenship, cyberbullying, evaluation, media literacy, middle school, conflict resolution, internet safety, mobile phone

## Abstract

**Background:**

*Screenshots* is an in-school curriculum that seeks to develop positive digital social skills in middle school students with the long-term goal of improving their health and well-being. The program imparts knowledge and teaches skills upon which young adolescents can build a set of beliefs and behaviors that foster respectful interactions, prosocial conflict resolutions, and safe and secure use of communication technology. Intervening in this way can improve young people’s mental health by limiting their exposure to cyberbullying and other forms of negative online interactions. This study reports on an evaluation of the *Screenshots* program conducted with seventh graders in a public school system in a midsized New England city.

**Objective:**

This study aims to determine the effectiveness of the *Screenshots* program in increasing participants’ knowledge about key concepts of digital citizenship and in shifting beliefs and intended behaviors to align with prosocial and safe online interactions. In addition, the study examines whether the program has varying effects on males’ and females’ conflict and bullying resolution strategies.

**Methods:**

This quasi-experimental evaluation was conducted in four middle schools in which one group of seventh graders received the *Screenshots* curriculum and another did not. Before and after the curriculum, all students completed a questionnaire that measured their knowledge of and beliefs about digital citizenship and related online behavioral concepts, their attitudes regarding strategies for stopping online bullying, and their intended online conflict resolution behaviors.

**Results:**

The sample included 92 students who received the curriculum and 71 students who were included in the comparison group. Pre- to postinstruction retention rates ranged from 52% (33/63) to 84% (21/25), varying by school and condition. The results showed an increase in knowledge about key curricular concepts for some students (*F*_1,32_=9.97; *P*=.003). In response to some individual items, students decreased their belief supportive of a negative online behavior (*F*_1,76_=9.00; *P*=.004) and increased their belief consistent with an online safety behavior (*F*_1,42_=4.39; *P*=.04) compared with the comparison group. Gender moderated the results related to conflict resolution, with males from one school reducing their endorsement of an aggressive option (*F*_2,40_=5.77; *P*=.006) and males from another school increasing their reported tendency to pursue a nonaggressive option (*F*_2,28_=3.65; *P*=.04). On average, participants reported learning something new from the classes.

**Conclusions:**

This study represents a rare evaluation of an in-school digital citizenship program and demonstrates the effectiveness of *Screenshots.* Students’ increased knowledge of key curricular concepts represents a foundation for developing future beliefs and healthy behaviors. Differences in how adolescent males and females experience and perpetrate online aggression likely explain the conflict resolution findings and emphasize the need to examine gender differences in response to these programs. Students’ high ratings of the relevance of *Screenshots*’ content reinforce the need for this type of intervention.

## Introduction

### Background

For decades, educators and other related stakeholders have developed curricula to teach media literacy; critical thinking; and associated skills necessary to analyze, decode, evaluate, and produce media messages [1]. There is considerable evidence that such programs have been successful in intervening on the impact of media on multiple social and health behaviors, including aggression [2], poor nutritional choices [3], and smoking [4]. As screen media transformed from a primarily receptive format with a few powerful creators to an interactive platform with countless, everyday authors, aspects of media literacy were applied to programs created to enhance *digital citizenship* and positive online social behaviors, including tolerance, respect, and empathy [5], that contribute to prosocial participation in online communities [6]. Although in-school cyberbullying interventions are regularly evaluated [7], assessments of other broader digital citizenship initiatives are very rare [8], with a single dissertation [9] identified in a recent review of this topic [10] and only a few published since then [11]. The purpose of this study is to examine the impact of *Screenshots*, a program designed by Media Power Youth that applies a health lens to digital citizenship. *Screenshots* strives to achieve digital wellness and improve young people’s mental health by encouraging respectful online behaviors, fostering prosocial conflict resolution, reducing cyberbullying, and interrupting negative peer pressure.

### Social and Digital Media and Adolescents’ Mental Health

There has been an increase in adolescent mental health issues (eg, depression and anxiety) and a decrease in psychological well-being (eg, self-esteem and life satisfaction) over the last two decades, which has occurred contemporaneously with a massive increase in smartphone adoption and social networking site use [12]. Meta-analyses and reviews of work linking digital media use in general and social media use in particular to adolescents’ mental health have found small, although significant, associations [13,14]. Many explanations for this potential link have been presented, ranging from social comparison to sedentary behaviors [14]. However, the consequences of social media use are not homogenous; the specific experiences young people have online will dictate the magnitude and direction of their impact. Youth can find positive peer support in social media [15] or they can experience increased depression [16] and risk for suicidal behaviors as a result of cyberbullying [17]. It would follow that being engaged with an online social network where interactions are guided by empathy and positive forms of conflict resolution would improve a young person’s mental health in ways similar to the positive effects of offline peer relationships [18]. Developing adolescents’ positive social behaviors and attitudes evident in digital citizenship would, therefore, encourage beneficial rather than detrimental interactions. Existing evidence is consistent with this approach, showing that digital citizenship is associated with lower levels of cyberbullying victimization and perpetration as well as a higher likelihood of being a helpful bystander [5].

### Designing In-School Initiatives

Media literacy education programs that are administered in schools as part of the standard school day take advantage of existing educational structures and norms, potentially increasing their impact over other delivery alternatives. Integrating this content into existing programming is a considerable challenge in most US schools, as their curricula are fairly inflexible. Working with health and art teachers has been successful when implementing educational initiatives in elementary school but might not work for middle school students who have a more rigid curriculum [19]. Incorporating digital citizenship messages across multiple middle school classes that already teach relevant information is potentially the strongest approach [8,20]; however, it is considerably difficult, as it requires coordination, training, and curricular development across faculty and disciplines. An alternate approach is to create digital citizenship curricula that are flexible enough to be taught by a number of different classroom teachers or other specialists while simultaneously meeting numerous core curricular goals.

### Theoretical and Conceptual Framework

Digital citizenship and related programs have at their core the goal of changing or shaping youth behavior, encouraging positive and respectful online behaviors [5] while preventing negative behaviors, such as cyberbullying [8]. The behavior change goals are well informed by existing models of human behavior. The Theory of Planned Behavior (TPB) illustrates how behavior can be altered by changing attitudes about a behavior, increasing a young person’s sense of self-efficacy to perform an action, and altering their perceived norms about the behavior [21]. TPB has been shown to be a useful framework for understanding and intervening on cyberbullying [22,23].

The Habits of Thought Model [24] provides an additional conceptualization of how changing beliefs and building a foundation of fact-based knowledge can encourage positive behaviors and constructive conflict resolution. On the basis of the work demonstrating the cognitive mediators of aggression [25], the Habits of Thought Model posits that the content of beliefs (what one thinks) and cognitive skills (how one thinks) shape an individual’s interpretation of an experience and their subsequent behavioral response. This model stems from Social Learning Theory and suggests that aggressive behaviors are learned and occur because an individual has underdeveloped abilities or existing biases in how they process social information [26]. Therefore, through education and practice that develop cognitive skills and alter related biases, aggressive behaviors can be reduced and positive behaviors can be increased. Interventions that follow this model train youth in effective conflict resolution strategies and alternate thought processes that encourage positive and empathetic choices for perpetrators of violence as well as victims and bystanders [27]. Students’ social processing skills are enhanced through exercises that help them avoid immediate negative responses, take time to assess social situations, and choose a prosocial solution. Therefore, the model is especially informative for programs seeking to foster prosocial online behaviors and digital citizenship. The anonymity available on the internet as well as the lack of immediate social consequences of negative behaviors contribute to antisocial behaviors online, even among young people who otherwise might not exhibit malicious behavior [28]. Furthermore, there are limited opportunities in online communication channels to express emotion and tone, making understanding the intent of online behaviors especially difficult. Considering how the online environment seems to foster a unique set of cognitive habits about aggression, digital citizenship programs that take a Habits of Thought approach can teach cognitive skills relevant to processing social information online and choosing positive behaviors and conflict resolution strategies in the digital space.

### Gender Differences in Aggression

Adolescent males and females perpetrate and experience aggression differently, both online and offline. In face-to-face settings, males tend to experience overt, externalized aggression, and females are more likely to be victims of relational, internalized aggression [29]. There is some evidence that these differences manifest in online aggression as well. Cyberbullying among males tends to be outwardly aggressive, with more threats of violence and direct insults, whereas females are more likely to post gossip with the intent to harm their peers [30,31]. Considering these differences, violence prevention interventions may be more effective if they target males and females separately using strategies aligned with the observed differences in aggression. In mixed-gender classrooms, such an approach is difficult to implement. The genders are, therefore, likely to react differently to lessons designed for and delivered in this setting. Understanding how males and females differ in their response to the cyberbullying and conflict resolution components of a digital citizenship education program can help inform the design of future curricula.

### Study Aim

The purpose of this study is to evaluate the effectiveness of *Screenshots* in improving students’ knowledge and changing beliefs and behaviors in the targeted areas of digital citizenship and to determine the extent to which students found the material engaging and relevant. The study also seeks to determine whether the program has varying effects on males’ and females’ conflict and bullying resolution strategies.

## Methods

### Curriculum Design

Media Power Youth is an educational nonprofit organization that provides media literacy and health curricula, youth programs, family education, and professional development for educators and staff of youth-serving organizations. Staff members and volunteers have experience in education, youth development, advertising, film making, and health advocacy. Media Power Youth directly facilitates programming and collaborates extensively with professionals across disciplines, including public health, technology, media, and research. Their programs have been developed as interventions to address youth risk factors, including substance use, violence, and mental health concerns, in diverse and underresourced populations.

Media Power Youth developed the *Screenshots* curriculum following the research, development, and implementation of elementary school media literacy curricula [19] and multiple out-of-school programs for youth and their families. The program was developed by a team of experienced educators who gathered and used feedback from teachers, social workers, and other professionals working with youth. The curriculum development and related activities were funded by the New Hampshire Department of Justice Office of the Attorney General, the Granite United Way, and the Norwin S. and Elizabeth N. Bean Foundation.

*Screenshots* is a nine-lesson, in-class, middle school curriculum that uses educational and behavior change strategies based on media literacy, TPB, and Habits of Thought to teach students how to think critically about the social media messages they create, receive, and disseminate. It delivers lessons that help youth manage online conflict, recognize how online behavior contributes to mental health problems, practice empathy, and explore the role of digital media in peer pressure that can lead to bullying and substance use. *Screenshots* helps students to gain knowledge about how online communication impacts behaviors, to develop beliefs about how best to promote online prosocial and healthy behaviors, and to cultivate the cognitive skills to actualize these new knowledge-based beliefs. It consists of three units with three lessons, each with each lesson designed to be implemented within a standard 45-minute class.

*Screenshots* aligns with the Common Core State Standards [32], International Society for Technology in Education Standards [33], and the New Hampshire Health Curriculum Standards [34], thereby increasing its usefulness in middle schools and ease of integration into existing curricula. Lessons feature suggested scripts for educators, hands-on student activities, role-play scenarios, and interactive notebooks for student reflections. The purpose of this multifaceted approach was to create an environment that supports multiple learning styles and student engagement. Podcasts are used as a springboard to introduce each unit. The curriculum concludes with a final project that enables students to share what they have learned by producing a form of media, an approach proven to enhance media literacy program effectiveness. Table 1 provides details about the lessons.

**Table 1:** Sample lesson plan objectives, activities, and messages from the Media Power Youth Screenshot curriculum.

Objectives			Activities		Messages
**Unit 1—Lesson 3: Healthy and Respectful Behavior Online**					
	Recognize that their online actions can have consequences that affect both their lives and the lives of others. Use the internet as an educational tool and a way to positively connect with others.	Online scenarios—students review online scenarios to stimulate discussion about their own behaviors. Online behavior quiz—students reflect on how their positive and negative behaviors impact other people. Stay safe—students review and add ways to stay safe online.		Online actions are part of who you are and can affect both your present and future. Students can show respect for themselves and others by acting appropriately online.	
**Unit 2—Lesson 2: Cyberbullying**					
	Recognize cyberbullying and empathize with the targets of cyberbullying. Recognize the key similarities and differences between in-person bullying and cyberbullying. Identify the strategies for dealing responsibly with cyberbullying.	Me or My Avatar—students create their own avatar. Online scenarios—students answer questions about how to react in certain online scenarios. Stop and STAND^a^—students learn a system to remember strategies for stopping and preventing cyberbullying.		The use of avatars might allow online users to say things or behave in ways they normally would not. Interacting with others in the digital world can sometimes lead to miscommunication. Online behaviors can be exaggerated, which can cause conflicts and result in situations escalating quickly.	
**Unit 3—Lesson 1: Digital Peer Pressure**					
	Identify what digital peer pressure is and the impact it can have on their choices and behaviors. Recognize images that are considered unhealthy and can lead to digital peer pressure. Take action to create more positive media messages.	Media images—students view images of drugs and alcohol and answer questions pertaining to them. Edit that Screenshot!—students learn techniques to help them handle peer pressure and apply the strategies to a novel scenario. Did you know?—Students view statistics about peer pressure.		Digital peer pressure can impact choices with regard to drug and alcohol use. Students can mitigate digital peer pressure by embracing instincts, deciding why they feel the way they do, identifying an appropriate action, and taking action by removing or editing unhealthy images.	

^a^STAND: Screenshot, Talk, Activate, Note, Defend.

### Curriculum Implementation

#### Overview

The curriculum was taught to seventh graders in four public middle schools in a midsized New England city. These schools were identified by district-level administrators as having a diverse student body as well as the resources and capacity necessary to implement the curriculum and follow the evaluation methods. Media Power Youth provided educator training before implementation for lead teachers in each school. At each school, teachers had multiple sessions of their class and chose one to be the curriculum group and another to be the no curriculum group. Although it was preferred that the first session of the day be designated as the curriculum group and the second session as the no curriculum group, the final decision of group assignment was left to the teachers’ discretion. The no curriculum group received standard instruction during the time in which the study was being conducted, and teachers were asked to implement the curriculum for the no curriculum group in the semester following the study. There was no schoolwide component of the curriculum, and no curriculum-related materials were posted in classrooms, thereby reducing the potential for cross-group contamination. Both groups completed pencil and paper questionnaires administered by their classroom teachers before and after the curriculum group received the lessons.

Although the curriculum was designed to be implemented as part of a designated course, each school had discretion in how they delivered the curriculum; as such, it was implemented in a variety of formats providing appropriate time blocks for delivering the materials, including during health classes, library sessions, and advisory periods. One of the four schools was dropped from the study as their implementation of the curriculum and evaluation deviated significantly from the others, resulting in a low retention rate (18/66, 27%) and introducing additional variables that prohibit comparison with the other schools. Teachers were asked to complete a fidelity reporting form following each lesson to track the administration of the lessons and to identify any problems with instruction. No major issues beyond what would be expected in a typical class were reported.

#### Procedures

Between October 2019 and January 2020, students at all four schools were given pre- and postinstruction questionnaires that obtained their demographic information and assessed their knowledge of key digital citizenship concepts as well as their beliefs about online behaviors, conflict resolution, and online bullying. The assessment was designed to be completed in approximately 20 minutes, so that, along with instructions and any potential questions, it could be completed during a single class. A complete list of the survey questions can be found in Multimedia Appendix 1. Participants’ two questionnaires were connected according to their responses to three questions: the last three letters of their last name, the day they were born, and the number of their home address (with instructions to cover various housing situations). The evaluation protocol was implemented by Media Power Youth staff and approved by the school board and school administrators, including an opt-out parental permission process standard for the participating district. The Boston Children’s Hospital Institutional Review Board designated the management and analysis of the anonymous data as exempt.

#### Sample Characteristics

Of the students who participated in the *Screenshots* program across all four schools, 218 completed both pre- and post -instruction assessments. Of the three schools included in the analyses (schools 1-3), 92 received the curriculum, and 71 served as a comparison. Table 2 provides demographic data by school. The students’ average age was approximately 12 years. Although the majority of students identified as a White person, there was considerable representation from other races and ethnic groups as well. Retention rates in the included schools differed by school and condition and varied from 52% (33/63) to 84% (21/25).

**Table 2:** Sample demographics (N=218).

Characteristic					School 1			School 2			School 3			School 4^a^
**Students per group, n (%)**														
	Curriculum			21 (48)			52 (61)			19 (56)			37 (67)	
	No curriculum			23 (52)			33 (39)			15 (44)			18 (33)	
**Questionnaire completion**														
	**Curriculum**													
		Postinstruction completion, n	21			52			19			37		
		Preinstruction completion, N	25			71			24			48		
		Questionnaire completion rate (%)	84			73			79			77		
	**No curriculum**													
		Postinstruction completion, n	23			33			15			18		
		Preinstruction completion, N	28			63			23			66		
		Questionnaire completion rate (%)	82			52			65			27		
Age (years), mean (SD)					12.1 (0.4)			12.2 (0.4)			12.2 (0.4)			12.2 (0.4)
Gender (female), n (%)					17 (38.6)			42 (49.4)			21 (61.8)			27 (50.0)
**Race, n (%)**														
	White (non-Hispanic)			24 (54.5)			47 (55.3)			20 (58.8)			32 (58.2)	
	Black (non-Hispanic)			2 (4.5)			7 (8.2)			3 (8.8)			4 (7.3)	
	Hispanic			4 (9.1)			17 (20.0)			5 (14.7)			13 (24.1)	
	Other and/or mixed race			14 (31.8)			14 (16.5)			6 (17.6)			5 (9.1)	

^a^Owing to implementation differences, school 4 was not included in any further analyses.

### Measures of Knowledge, Beliefs, and Behaviors

#### Core Curriculum Knowledge

To assess the acquisition of the concepts taught in the *Screenshots* curriculum, we created nine multiple-choice, general knowledge questions based on the three focus areas covered in the program: digital citizenship, online conflict resolution, and media and peer pressure. For example, participants were asked, “Which of the following should you NOT share online?” with the following answer options: “a video of an activity you do,” “your first name,” “your phone number” (correct answer), “sports/activities you play.” All questions had four answer choices and one answer that best aligned with the curriculum. The total number of questions answered correctly were summed and used for the data analysis.

#### Digital Citizenship Beliefs

Key beliefs targeted by the curriculum and reflective of an alignment with digital citizenship concepts were assessed through seven questions created for the evaluation. Questions asked participants to rate on a 5-point Likert scale how strongly they agreed or disagreed with specific beliefs related to the acceptability, impact, and risk of different online behaviors and actions, including, for example, “My online posts are my own thoughts and do not impact other people.” Separate analyses were conducted for each question.

#### Digital Citizenship Behaviors

Students were asked to report on their own prosocial online behaviors that would be indicative of well-developed citizenship. Four questions were adapted from a Department of Justice funded internet safety education outcome survey [35]. Participants reported how much each statement sounded like them using a five-point scale that ranged from *not at all* to *very much*. Items included their tendency to end arguments and drama online as well as other indicators of respectful behaviors and were analyzed individually.

### Measures of Aggression

#### Conflict Resolution

Participants were given two scenarios of potential conflict and six different strategies for responding to the situations. The first scenario dealt with rumors being spread around school through negative posts on social media about a participant’s friend. The second detailed a scenario in which a photo of *you* that *you* did not like was taken and shared on social media. Participants rated on a five-point Likert scale how likely (from *would definitely* to *would definitely not*) they were to use each of the six different strategies categorized as verbal problem solving, avoidance, seeking help from a trusted adult, information seeking or understanding the situation, verbal aggression, and overt aggression. This approach is based on an offline conflict resolution scale [25], with scenarios adapted from previous media literacy program evaluations and the internet safety education outcome survey [5,20,35].

#### Strategies for Stopping Online Bullying

Participants were asked to imagine that they were being bullied in an online group and to rate, on a five-point Likert scale, the effectiveness of each strategy to stop the bullying (eg, telling the bully to stop bothering you and talking to a trusted adult or family member about the bullying).

#### Responsiveness and Relevance

Four responsiveness and three relevance questions derived from previous media literacy evaluations [19,36] were given to participants who received the curriculum on the postinstruction questionnaire. The questions asked participants to rate how strongly they agreed or disagreed (five-point Likert scale) with statements about their engagement and enjoyment of the curriculum as well as the importance of the program content.

### Analysis Plan

Mixed method (within- and between-subjects) analyses of variance were performed to compare the two conditions in their pre- and postinstruction responses with a time-by-condition interaction, indicating a difference in pre-post change between the two groups. Given that schools differed substantially in their implementation of the curriculum, we conducted analyses separated by school. To examine gender differences in responses to the conflict and bullying resolution components of the curriculum, we added a three-way time-by-condition-by-gender interaction to analyze questions related to these concepts. Along with our significant findings of *P*<.05, we presented results with significance values of *P*<.10 and considered the effect sizes (partial *η^2^*) of the findings as we interpreted the results. We interpreted these findings when they were supportive of or similar to the significant findings. The aim of this approach was to avoid discarding results that may not reach statistical significance but may still be meaningful for those implementing and designing digital citizenship programs.

## Results

### Core Curriculum Knowledge

Table 3 shows the results of the mixed analyses of variance for the core curriculum knowledge questions. For school 3, there was a significant increase in questions answered correctly after receiving the *Screenshots* curriculum. For school 2, there was some evidence, although only at the level of *P*=.08, that the curriculum and comparison groups differed in the same way as in school 3.

**Table 3:** Results of the mixed analyses of variance for the core curriculum knowledge questions.

School and group		Group pretest, mean^a^ (95% CI)	Group posttest, mean^a^ (95% CI)	*F* test (*df*)		*P* value		Partial *η^2^*	
**School 1**					0.001 (1,42)		.97		0.00
	Curriculum	5.76 (5.14-6.39)	6.05 (5.29-6.81)						
	Comparison	5.04 (4.45-5.64)	5.35 (4.62-6.08)						
**School 2**					3.17 (1,83)		.08		0.04
	Curriculum	5.02 (4.60-5.44)	5.37 (4.93-5.80)						
	Comparison	5.15 (4.63-5.68)	4.85 (4.30-5.40)						
**School 3**					9.97 (1,32)		.003		0.24
	Curriculum	4.84 (4.11-5.58)	6.00 (5.43-6.57)						
	Comparison	5.27 (4.44-6.10)	4.93 (4.30-5.57)						

^a^The mean number of correct responses to the nine multiple-choice questions assessing participants’ understanding of core digital citizenship concepts.

### Digital Citizenship Beliefs

In school 2, students increased their mean beliefs regarding the three questions to be better aligned with digital citizenship concepts. They disagreed more strongly with the statement, “If my friends and I need a laugh, it’s ok for us to post a funny and embarrassing picture of someone else online” (pretest=4.41; posttest=4.54; *F*_1,76_=9.00; *P*=.004; *η_p_^2^*=0.106). Although not reaching the level of significance, other results showed differences in the same direction: “My online posts are my own thoughts and do not impact other people” (pretest=3.40; posttest=3.65; *F*_1,77_=3.25; *P*=.08; *η_p_^2^*=0.04) and “It’s ok to share my online passwords with a friend that I trust” (pretest=3.96; posttest=4.02; *F*_1,76_=2.79; *P*=.10; *η_p_^2^*=0.035). The comparison group increased their agreement with each of these statements. At school 1, students significantly increased their agreement with the statement, “If there’s a video or picture that I don’t want lots of people to see, setting my profile to ‘private’ will keep it secret” (pretest=3.52; posttest=3.33; *F*_1,42_=4.39; *P*=.04; *η_p_^2^*=0.095), whereas the comparison group decreased their agreement.

### Conflict Resolution

With regard to the conflict resolution scenarios, a condition-by-time-by-gender interaction was tested to determine whether the effects of the curriculum differed between males and females. For the scenario describing someone posting a photo of *you* that *you* do not like, males in the *Screenshots* group of school 1 decreased their endorsement of an aggressive response, “Tell them they better take the photo down or you’ll get back at them” (pretest=3.36; posttest=2.36; *F*_2,40_=5.77; *P*=.006; *η_p_^2^*=0.224), whereas their peers in the comparison group increased their endorsement (pretest=2.69 and posttest=2.81). Females in the program slightly increased their endorsement of this aggressive behavior (pretest=2.50 and posttest=3.00), and females in the comparison group decreased their endorsement (pretest=3.00 and posttest=2.00). At school 3, males increased (pretest=2.67 and posttest=3.50) and females decreased (pretest=3.83 and posttest=3.58) their reported likelihood of seeking additional information about the behavior (“asking the person why they posted the photo online”; *F*_2,28_=3.65; *P*=.04; *η_p_^2^*=0.207).

### Strategies for Stopping Bullying

In school 3, a significant three-way interaction indicated differences between how males and females endorsed the effectiveness of the strategy of “standing up to the bully by fighting back.” Males who received the curriculum showed an increased endorsement (pretest=3.00 and posttest=3.17), whereas males in the comparison group (pretest=3.20 and posttest=2.40) showed a decreased endorsement (*F*_3,29_=4.399; *P*=.02; *η_p_^2^*=0.233). Females in both the *Screenshots* (pretest=2.54 and posttest=2.62) and comparison groups (pretest=1.88 and posttest=2.50) increased their endorsement of this strategy. The results for other questions and at other schools did not reach significance but may indicate at least weak evidence for differences. Among students who received *Screenshots* in school 2, males increased their mean endorsement of talking to a trusted adult as a method to stop bullying (pretest=3.75 and posttest=4.07), whereas those in the comparison group showed a mean decrease (pretest=3.93; posttest=3.79; *F*_2,77_=2.56; *P*=.08; *η_p_^2^*=0.062). For females, the curriculum group decreased their assessment of the strategy (pretest=4.32 and posttest=4.27), whereas the comparison group increased their assessment of the strategy (pretest=3.35 and posttest=3.88).

### Responsiveness and Relevance

Table 4 shows the mean responses of students to each of the four responsiveness and three relevance questions. Students responded fairly neutrally to the program, with scores near 3 on a five-point scale for items about their interest in and enjoyment of the classes. Relevance items received higher ratings, indicating that the students regularly agreed that they learned something new from the class and that other students should take the course.

**Table 4:** Results of the responsiveness and relevance questions.

Question^a^	School 1, mean (SD)	School 2, mean (SD)	School 3, mean (SD)
The classes were interesting	2.92 (1.09)	3.21 (0.86)	2.55 (1.14)
I got easily distracted during the classes	3.46 (1.14)	2.95 (0.99)	3.35 (0.98)
I enjoyed the classes	2.96 (1.24)	3.27 (1.04)	2.83 (1.03)
The classes were boring	3.23 (1.24)	2.85 (0.99)	3.48 (1.16)
I learned something new from these classes	3.62 (0.94)	3.89 (1.01)	3.35 (1.52)
Other kids my age should take these classes	3.38 (1.20)	3.88 (0.99)	3.22 (0.95)
I learned something from these classes that I will use in my own life	3.44 (1.08)	3.82 (1.04)	2.91 (1.31)

^a^Results represent the mean (SD) of seven postassessment questions given to participants who received the *Screenshots* curriculum, with response choices of 1=strongly disagree and 5=strongly agree.

## Discussion

### Principal Findings

The aim of this study is to evaluate the effectiveness of the *Screenshots* curriculum in improving students’ knowledge and changing their beliefs and intended behaviors around positive online social interactions, conflict resolution, and other aspects of digital citizenship. In one of the three schools included in the analyses, the students in the program increased their knowledge relevant to the lessons from the pretest to the posttest. Although the evidence was weaker, a similar change was observed at a second school. There was also some evidence showing that students’ beliefs changed to be less supportive of unkind online behavior and supportive of privacy and safety, including understanding that privacy settings will protect a profile.

The results for conflict resolution and bullying differed by gender and were somewhat inconsistent in direction. At one school, males in the program decreased the likelihood that they would use verbal aggression and increased their endorsement of the more prosocial conflict resolution approach of seeking additional information about another person’s behaviors. Females at the same school showed the opposite results; they increased their likelihood of using verbal aggression and decreased their likelihood of seeking additional information. Males in the *Screenshots* group increased their endorsement of strategies to disrupt bullying that were both overly assertive (standing up to the bully and fighting back) and, to a lesser extent, prosocial (seeking adult help and peer involvement).

### Digital Citizenship Knowledge, Beliefs, and Behaviors

*Screenshots* was most successful at improving participants’ knowledge of online behaviors and healthy communication practices consistent with digital citizenship. In addition, students self-reported that they had learned something new in the curriculum. Such a change is at the core of the program and can serve as a foundation for a belief and behavior system that is based on information rather than erroneous ideas. College students have been found to have limited knowledge regarding good digital citizenship, and experience using the internet does not seem to compensate for this shortcoming [37]. Programs such as *Screenshots* that target young adolescents are necessary to fill this educational gap.

There was some, although limited, evidence that participants changed their beliefs in response to the program, but they did not change their immediate behaviors. However, this is not surprising. Experienced educators in this field have observed that students often demonstrate an understanding of appropriate online behaviors, but they do not actually follow through in practice [10]. Furthermore, altering strongly held beliefs in young adolescents through direct education, especially in a topic in which they have a sense of proficiency, is particularly difficult as they are moving from adults to peers as their primary source of social information [38]. Even given this challenge, *Screenshots* encouraged students in one school to hold beliefs more consistent with the idea that it is not okay to post embarrassing pictures of other people or to share online passwords. These results demonstrate potential changes relevant to the two roles of cyberbullying: sharing embarrassing pictures is a common tool for perpetrators of cyberbullying and sharing online passwords may increase the risk of victimhood [39,40]. Although these findings represent a modest success of *Screenshots,* long-term interventions or programs with additional booster lessons over multiple school years would likely be necessary to help students more fully assimilate new knowledge from the curriculum and ensure it is translated into behaviors. Other educational approaches should also be explored, such as project-based learning that encourages students to guide their own investigation into the topic and develop solutions, thereby becoming more connected to the content [41].

### Gender Differences

Although not universal, violence prevention programs have been shown to operate differently for males and females. In one such intervention, males were found to reduce their externalizing behavior problems and bullying [42], whereas females showed better outcomes related to relational aggression [43]. Our results reflect these findings to some extent, with some males decreasing their endorsement of a verbally aggressive behavior choice. Other males, however, increased their likelihood of using prosocial problem solving to address a potential conflict, but females did not. Males likely perceive the role of aggression in online conflict very differently from females and experience it in a different context, including it being modeled as a solution in violent video games [44]. Considering that the Habits of Thought Model was originally developed as an approach to reduce aggression in males, the approaches used by *Screenshots* may be somewhat more effective for males. It is worth noting that we also observed that males increased their endorsement of *Standing up to the bully by fighting back online*. The program may have encouraged males to support their friends, and it was the helping, rather than the subtle aggression, that they were responding to in these responses. Similarly, females slightly increased their likelihood to *Tell them they better take the photo down or you’ll get back at them.* Again, the females in the program may be focusing on the verbal assertion rather than the retaliation component of this behavior. Regardless, future research should determine whether this observed boomerang effect is replicated or unique to this evaluation. Furthermore, additional work is necessary to fully understand the moderating role of gender on the effects of online conflict resolution interventions, especially the potential for varying impacts among sexual minority youth.

### Study Limitations

This study represents one of the very few empirical evaluations of an in-school digital citizenship program. However, the findings of this study should be interpreted within the context of some important limitations. School district requirements led to the study’s focus on seventh graders rather than the full middle school age range, resulting in a much smaller sample than would be ideal. The use of a single grade level as well as the constrained geographic location of schools limit the generalizability of the findings. Furthermore, differences across the schools in programming and daily class structures limited our ability to maintain identical implementation techniques at each site and contributed to various administration challenges. As a result, we were unable to use data from all schools and could not pool data across schools. Such challenges are an inherent aspect of research conducted in school settings where teachers must first meet and adjust to structural, administrative, and student needs before attending to the needs of a research study. Together, these limitations reduced our intended sample size and our ability to detect small effects of the curriculum, meaning that some important differences may not have risen to the level of significance. Presenting and discussing some findings that only reached the significance level of *P*<.10 is an additional limitation to our approach, as it increases the likelihood that we are making claims based on chance findings. Nevertheless, the in-school, quasi-experimental design strengthens the approach of this study, making it a rare example of a real-world evaluation of this type of program.

### Conclusions

It is the goal of our schools to prepare children for their future. The COVID-19 pandemic has dramatically altered that future in that vestiges of full-time online instruction, technology-mediated communication, and internet-based social systems that have dominated this time will inevitably persist well beyond the COVID era. Skills related to online conflict resolution and positive behaviors that were once considered niche abilities are now essential for overall mental health. *Screenshots* and other similar programs that teach these skills fill a critical educational need in today’s world. This study demonstrated that *Screenshots* improved seventh graders’ knowledge and holds some promise in changing beliefs related to digital citizenship and the prosocial behaviors that support mental health. Students recognized the need for this program, indicating that they learned something new that would be relevant to other children. Hopefully, this work will inspire additional school-based research in this area, so that educators can be armed with curricula known to be effective in encouraging positive online behaviors and subsequently improving mental health and well-being.

## Appendix

Multimedia Appendix 1 Survey measures of the <italic>Screenshots</italic> evaluation.

## Abbreviations

TPB: Theory of Planned Behavior
