# Supplementary material for: Evaluating a Middle-School Digital Citizenship Curriculum (Screenshots): Quasi-Experimental Study
Source: JMIR Ment Health. 2021 Sep 15;8(9):e26197. doi: 10.2196/26197 (PMC8482248; doi:10.2196/26197)
Supplement: Multimedia Appendix 1 [file mental_v8i9e26197_app1.pdf]

Appendix: *Screenshots* Survey Measures

1. This is a Multimedia Appendix to a full manuscript published in JMIR Ment Health. For full copyright and citation information see http://dx.doi.org/10.2196/26197.

General Core Curriculum Knowledge Questions

Please select one answer choice for each of the following questions.

Which of the following should you NOT share online?

- [ ] Video of an activity you do.
- [ ] Your first name.
- [x] Your phone number.
- [ ] Sports/activities you play.

Which of the following is something that could make you less safe online?

- [ ] Communicating with a classmate online.
- [ ] Visiting YouTube or other similar video site.
- [ ] Sharing a password with your parent.
- [x] Letting a friend use your social media account.

Which of the following is true about your digital footprint?

- [ ] You have total control over it.
- [ ] It doesn't last long.
- [x] It includes everything you have done on the Internet.
- [ ] It can only have negative effects on you.

Which of the following is LEAST important to consider before posting, sharing, or sending something online?

- [x] Is it something others will agree with?
- [ ] Is it helpful?
- [ ] Is it true/ factual?
- [ ] Is it a nice thing to say?

Which of the following statements about online interactions is TRUE?

- [ ] People always act the same way online as they would offline.
- [x] When you post something online, it can become permanent even if you delete it.
- [ ] You should only take action in an online argument if you are involved.
- [ ] Online interactions make people feel more confident and protected, which is always a good thing.

Choose the answer that BEST finishes the following sentence. Empathy is…

- [x] thinking about and understanding how others might feel.
- [ ] including peers in social activities.
- [ ] only possible in face-to-face interactions.
- [ ] being welcoming to all kinds of people.

Choose the statement about digital peer pressure that is TRUE:

- [ ] Digital peer pressure is more common among younger students.
- [ ] Digital peer pressure is when other people say mean things about your posts.
- [ ] Digital peer pressure usually encourages healthy behaviors.
- [x] Digital peer pressure can make you feel like you should try drugs or alcohol.

Which of the following is the MOST IMPORTANT question you should ask to help find the Hidden Story behind an online image or media message online?

- [x] What types of consequences were left out?
- [ ] How might this image alter your digital footprint?
- [ ] Who are the people being shown?
- [ ] How is the image or message using digital peer pressure?

Which of the statements below is FALSE?

- [x] Media messages are most effective when they are based on facts instead of emotions.
- [ ] Media messages are designed to persuade, inform, or entertain.
- [ ] Media messages are influenced by the thoughts of the person creating the media.
- [ ] Media messages can make people believe situations are more common than they really are.

Strategies to Stop Online Bullying

Imagine that you are being bullied in an online group. How well do you think each of these strategies would work to stop the bullying?

	Definitely would NOT work	Probably would NOT work	Might work/might not work	Probably WOULD work	Definitely WOULD work
Talking to a trusted adult or family member about the bullying.	☐	☐	☐	☐	☐
Standing up to the bully by fighting back online.	☐	☐	☐	☐	☐
Telling the bully to stop bothering you.	☐	☐	☐	☐	☐
Getting your friends to tell everyone in the group that bullying is not OK.	☐	☐	☐	☐	☐

Online Citizenship Belief Questions

Select the answer that best describes how you agree with the following statements:

	Strongly disagree	Disagree	Neither disagree nor agree	Agree	Strongly agree
If there's a video or picture that I don't want lots of people to see, setting my profile to “private” will keep it secret.	☐	☐	☐	☐	☐
My online posts are my own thoughts and do not impact other people.	☐	☐	☐	☐	☐
People behave pretty much the same online as they do offline.	☐	☐	☐	☐	☐
Having empathy for other people can improve communication in online communities.	☐	☐	☐	☐	☐
Seeing images of smoking and drinking online makes people more likely to smoke or drink.	☐	☐	☐	☐	☐
It's ok to share my online passwords with a friend that I trust.	☐	☐	☐	☐	☐
If my friends and I need a laugh, it's ok for us to post a funny and embarrassing picture of someone else online.	☐	☐	☐	☐	☐

Online Citizenship Behavior Questions

Please select the response that is most like you for the following statements:

	Not at all like me	Not much like me	Some-what like me	Mostly like me	Very much like me
When I am online, I try to end arguments or dramas when they develop.	☐	☐	☐	☐	☐
I think about making sure that things I say and post online will not be something I regret later.	☐	☐	☐	☐	☐
I am careful about how I say things online, so they don't come across the wrong way.	☐	☐	☐	☐	☐
If I saw a fight happening at school, I would post about it as soon as possible to help my friends stay updated.	☐	☐	☐	☐	☐

Conflict Resolution Questions (scenario 1)

Please read the following scenario:

Someone at school is spreading rumors and making mean comments about someone you know by posting comments on Instagram. Other kids have started to join in.

How likely is it that you would do each of the following?

	Would definitely DO this	Would probably DO this	Might or might not do this	Would probably NOT do this	Would definitely NOT do this
Tell the person who was causing the problem to stop.	☐	☐	☐	☐	☐
Ignore or avoid the person who was causing the problem.	☐	☐	☐	☐	☐
Talk with an adult in your family or a teacher at school about the problem.	☐	☐	☐	☐	☐
Post some questions to try to figure out what happened and why people are acting this way.	☐	☐	☐	☐	☐
Help your friend by posting mean comments right back at the other kids.	☐	☐	☐	☐	☐
Start spreading rumors about the other kid in school and online.	☐	☐	☐	☐	☐

Conflict Resolution Questions (scenario 2)

Please read the following scenario:

A classmate takes your picture at school and you don't like the way you look. Later that night you see the photo posted on another student's Instagram story.

How likely is it that you would do each of the following?

	Would definitely DO this	Would probably DO this	Might or might not do this	Would probably NOT do this	Would definitely NOT do this
Tell that person that you want them to take the photo down.	☐	☐	☐	☐	☐
Tell them they better take the photo down or you'll get back at them.	☐	☐	☐	☐	☐
Ask the person why they posted the photo online.	☐	☐	☐	☐	☐
Take a bad photo of the person without them knowing and post it on your profile.	☐	☐	☐	☐	☐
Do nothing and ignore the situation.	☐	☐	☐	☐	☐
Tell a teacher or other adult.	☐	☐	☐	☐	☐

Responsiveness and Relevance Scale (on post-test for curriculum participants only)

Please tell us what you think about ALL the media classes you had:

	Strongly disagree	Disagree	Neither agree or disagree	Agree	Strongly agree
The classes were interesting.	☐	☐	☐	☐	☐
I got easily distracted during the classes.	☐	☐	☐	☐	☐
I enjoyed the classes.	☐	☐	☐	☐	☐
The classes were boring.	☐	☐	☐	☐	☐
I learned something new from these classes.	☐	☐	☐	☐	☐
Other kids my age should take these classes.	☐	☐	☐	☐	☐
I learned something from these classes that Iwill use in my own life.	☐	☐	☐	☐	☐
